# High Thermal Stability, High Tensile Strength, and Good Water Barrier Property of Terpolyester Containing Biobased Monomer for Next-Generation Smart Film Application: Synthesis and Characterization

**DOI:** 10.3390/polym12112458

**Published:** 2020-10-23

**Authors:** Jaemin Jeong, Fiaz Hussain, Sangwon Park, Soo-Jung Kang, Jinhwan Kim

**Affiliations:** 1 Department of Polymer Science and Engineering, Sungkyunkwan University, 2066, Seobu-ro, Jangan-gu, Suwon, Gyeonggi 16419, Korea; jjm123kr@gmail.com (J.J.); fiazravian@gmail.com (F.H.); bluswon@gmail.com (S.P.); soojung@skku.edu (S.-J.K.); 2 Institute of Advanced Materials, Bahauddin Zakariya University, Multan 60800, Pakistan

**Keywords:** bio-based, copolyester, dimensional stability, flexible optical devices, uniaxial stretching, birefringence, and barrier properties

## Abstract

This research synthesizes novel copolyester (PCITN) containing biobased isosorbide, 1,4-cyclohexandimethanol, terephthalic acid, and 2,6-naphthalene dicarboxylic acid and characterize its properties. The PCITN copolyester was extruded into film, and its performance properties including: tensile strength, Young’s modulus, thermal, dimensional stability, barrier (water barrier), and optical (birefringence and transmittance) were analyzed after uniaxial stretching. The films have higher *T*_g_, *T*_m_, dimensional stability, and mechanical properties than other polyester-type polymers, and these performance properties are significantly increased with increasing stretching. This is due to the increased orientation of molecular chains inside the films, which was confirmed by differential scanning calorimetry (DSC), X-ray diffraction (XRD), and birefringence results. Good water barrier (0.54%) and lower birefringence (△n: 0.09) of PCITN film compared to poly(ethylene terephthalate) (PET), poly(ethylene 2,6-naphthalate) (PEN), and polyimide (PI) films, used as conventional substrate materials for optical devices, make it an ideal candidate as performance material for next-generation flexible devices.

## 1. Introduction

Biobased polymers have gained the attention of scientists due to the rapid depletion of fossil fuels and increasing environmental pollution [1,2,3,4]. With the advancement and innovation of biotechnology and chemical industry, a wide range of biobased monomers such as isosorbide (ISB) [5], lactic acid [6], tannic acid [7], 2,5-furandicarboxylic acid [8], succinic acid [9], etc. have been developed and are being used widely for the development of biobased polymers. However, their poor mechanical and thermal properties limit their industrial applications for engineering polymers. 

Among various biomass monomers, isosorbide (1,4:3,6-dianhydrohexitol; ISB) is well known due to its superior performance properties. It is derived from glucose, and its bifunctional hydroxyl groups facilitate the condensation or addition polymerization. Its unique rigid structure and chirality significantly improve the glass transition temperature (*T*_g_) and transparency of the resulting polymers. Many efforts have been done to develop the ISB-based polyesters [10], epoxies [11], polyurethane [12], and polycarbonates [13,14], which are not only environmentally friendly but also have superior thermal and optical properties. However, it is very difficult to get a high molecular weight product using ISB alone due to its low reactivity and degradability during the polymerization process.

Poly(ethylene terephthalate) (PET) and poly(ethylene naphthalate) (PEN) are well known conventional homopolyesters. High crystallizability, low *T*_g,_ and inferior barrier properties of PET act as obstacles in its commercial applications, especially at high temperatures (˃100 °C). PEN has good thermal stability, however, expensive raw materials used for its synthesis, high birefringence, and necking of the film during stretching limit its commercial applications. To replace the conventional polymeric substrate for flexible electronics, materials need to offer ease of processing, high *T*_g_, good dimensional and thermal stability, good optical, and moisture barrier properties. The properties of the polymers can be significantly improved by blending [15]. The performance properties of the polyester-based composites are significantly higher than copolyesters [16]. However, polyester-based composites are not suitable for the application where transparency and flexibility are required. So, it would be of great interest to develop a biobased transparent and flexible copolyester for versatile industrial applications.

This research focuses on the synthesis of terephthalic acid (TPA), 2,6-naphthalene dicarboxylic acid (NDA), 1,4-cyclohexanedimethanol (CHDM), and isosorbide (ISB)-based copolyester that has high *T*_g_ and good mechanical, thermal, and barrier properties, even with low ISB content compared to conventional polyesters. It is important to note that the low reactivity of ISB prevents the obtaining of high molecular weight polymer products, which limits the commercial application of polymers as engineering plastics [17,18,19]. To overcome this problem, CHDM was incorporated to facilitate between TPA, NDA, and ISB. Finally, PCTIN copolyesters containing 25 mole% of ISB (feed ratio) with high weight average molecular weight (Mw: 68,900 g/mol) and *T*_g_ (121 °C) were successfully synthesized by two-step melt polymerization. Novel PCITN copolyester film was fabricated using a melt extrusion process to extend their commercial applications and analyze their performance properties. We significantly improved the thermal, mechanical, dimensional stability, and barrier properties, which is the weak point of bio-based polymers, by utilizing additional monomers: CHDM, TPA, and NDA.

In this study, the fabrication and characterization of biobased novel PCITN film are being reported to explore its versatile industrial applications such as a smart film. The fabricated semi-crystalline film was uniaxially stretched (λ1–λ4) in machining direction (MD) at 150 °C. The influence of stretching on thermal, mechanical, dimensional stability, optical, and barrier properties was studied. Analyzed performances were found to increase linearly with increasing stretching, which is due to increased molecular orientation in the stretched films. It is obvious from the results that based on the unique performance properties of PCITN compared to PET, PEN, and Polyimide (PI) [20,21], this novel material has the potentional to be used as a performance material in next-generation flexible electronic devices. 

## 2. Materials and Methods

### 2.1. Materials

TPA (99.9%), ISB (99.8%), and CHDM (99.8%) with 70 mole% trans-isomers were purchased from SK Chemicals (SKC; Suwon, Korea). NDA (99.8%) was purchased from BASF (Ludwigshafen, Germany). The ultra-pure (99%) catalyst (titanium-N-butoxide (TNBT) and thermal stabilizer (phosphorous acid) were used as received from Sigma Aldrich (Soul, South Korea). The high-purity monomers were used as received. High purity (99.9%) solvents, like deuterated chloroform (CHCl_3_-d) (Sigma Aldrich, Soul, South Korea), deuterated trifluoroacetic acid (TFA-d) (Sigma Aldrich, Soul, South Korea), and o-chlorophenol (Sigma Aldrich, Soul, South Korea) used for the characterization of specimens, were used as received without any further purification.

### 2.2. Synthesis of Copolyester Followed by Solid-State Polycondensation (SSP)

#### 2.2.1. Copolyester Synthesis

**Scheme 1:**
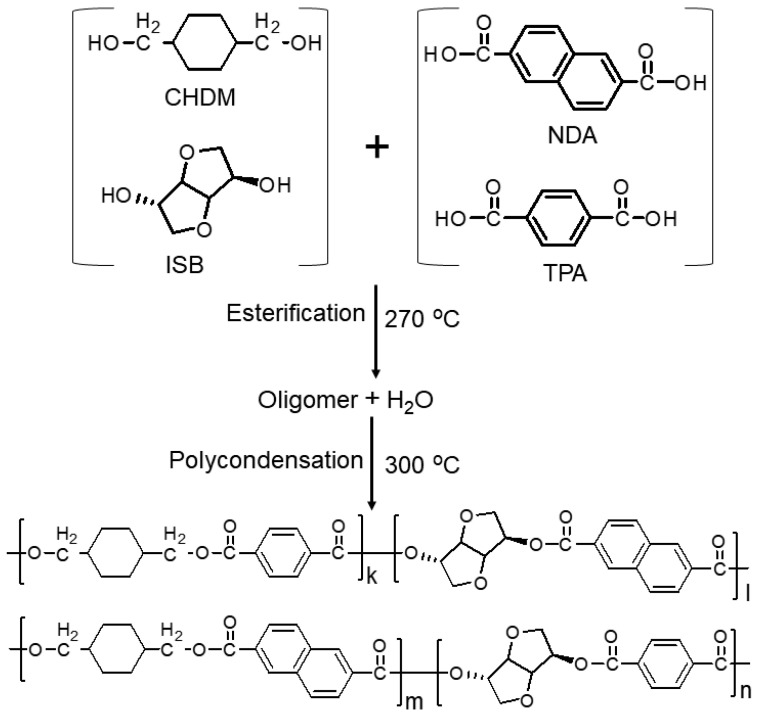
The schematic diagram for the melt polymerization of PCITN copolyester.

PCITN copolyester was melt polymerized using two different 5 L batch reactors of the pilot-scale melt polymerization reactor [22]. For esterification, the residual oxygen was removed from the reactor by purging pure nitrogen (N_2_). All the chemicals: TPA (249 g, 1.5 moles), NDA (973 g, 4.5 moles), CHDM (1089 g, 6.8 moles), ISB (331 g, 2.3 moles) titanium butoxide catalyst (3.906 g; 300 ppm relative to the total expected weight of the product), and phosphorous acid stabilizer (0.4850 g; 100 ppm relative to the total expected weight of the product) were charged into the esterification reactor at a molar ratio of diacid:diol = 1.0:1.5 for PCITN. The temperature of the reaction mixture was increased slowly up to 270 °C and held at this temperature until the completion of the esterification reaction. At this stage, water (by-product) generated during esterification reaction has been distilled out. After, this product was transferred into the polycondensation reactor. For transesterification, the temperature was gradually increased to 300 °C, the vacuum was reduced to 0.5 Torr, and these conditions were maintained until the completion of the reaction. Torque meter value (Nm) was used to determine the end-point of the reaction. The stirring speed was reduced gradually as the degree of polymerization increased. The polycondensation reaction continued isothermally at 90 RPM, 60 RPM, and 30 RPM until the torque value reached 1.6 Nm at each RPM. Finally, the reactor pressure was returned to atmospheric pressure by purging N_2_ gas to avoid the oxidative degradation of the polymer. The resulting PCITN copolyester was extruded from the reactor, quenched, and pelletized. The schematic diagram for the melt polymerization of PCITN is shown in Scheme 1.

Solid-state polycondensation (SSP) is an important step of the polymer industry as it significantly increases the molecular weight of synthesized polymers making them suitable for a wide range of commercial applications as an engineering plastic [23,24,25]. SSP of synthesized PCITN copolyester was also carried out at optimized conditions (Temperature and time) under the nitrogen atmosphere. The synthesized PCITN copolyester was thoroughly characterized for its chemical structure, thermal, and degradation behavior after SSP at 250 °C for 20 h.

#### 2.2.2. Film Fabrication and Uniaxial Stretching

The extrusion grade PCITN copolyester having a high molecular weight (*M*_w_: 68,900) and intrinsic viscosity (IV: 0.77) was successfully fabricated into a film using the melt extrusion process at 290 °C. The randomly-oriented film was successfully fabricated (thickness; 550 µm and width; 18 cm) and wound up on a cold stainless-steel winder roll (25 °C). 

As synthesized, randomly-oriented film specimens were preheated for 25 min before tensile stretching and then uniaxially cold drawn (at 150 °C) in MD using a tensile stretching machine with a speed of 1.3 mm/sec. To freeze the orientation of aligned polymeric chains obtained during drawing, the stretched films were cooled down to room temperature using cold air without removing the stress. The films with different draw ratios (λ) (1~4) were thus obtained and they were characterized for their thermal, mechanical and thermal degradation behavior; dimensional stability; and optical and barrier properties.

### 2.3. Characterization

The IV (dL/g) of PCITN copolyester was measured in the o-chloroform (OCP) solvent (15 g/15 mL) using an automated Ubbelohde viscometer (no. 1C) at 30 °C. The number average molecular weight (*M*_n_), weight average molecular weight (*M*_w_), and polymer dispersity index (PDI) were measured by gel permeation chromatography (GPC, Agilent, Santa Clara, California, United States) using m-cresol as a mobile phase at a velocity of 0.7 mL/min. The separation was performed with two Shodex LF804 columns at 100 °C equipped with a Malvern TDA 305 refractive index detector. The sample solutions were filtered using a polytetrafluoroethylene microporous membrane (Merck Millipore, 0.45 μm pore size) before injection. *M*_n_, *M*_w_, and PDI (*M*_w_/*M*_n_) were determined from universal calibration with polystyrene standards.

For the actual chemical composition of synthesized copolyester, proton nuclear magnetic resonance (^1^H-NMR, Bruker Cooperation, Billerica, Massachusetts, United States) spectra were obtained at 25 °C by a Unity Inova 500NB High Resolution 500 MHz NMR Console. Deuterated chloroform (CHCl_3_-d) and deuterated trifluoroacetic acid (TFA-d) (1:1) were used as solvent and tetramethylsilane (TMS) as an internal standard and as reference for chemical shifts (parts per million, ppm). Chemical shifts were expressed in ppm (parts per million) relative to an internal standard (TMS).

Thermal properties of polymer pallets were analyzed by differential scanning calorimeter (DSC Q20, TA instruments, New Castle, Delaware, USA) thermograms. About 8–10 mg of the sample was melted at 300 °C and then quenched to 40 °C at a cooling rate of 200 °C/min. Then, the temperature was raised from 40 to 300 °C at a heating rate of 10 °C/min under N_2_ atmosphere. Thermal decomposition behavior was analyzed by thermo-gravimetric analyzer (TGA Q50, TA instruments, New Castle, Delaware, USA) thermograms. For this, the sample placed in the alumina pan of TGA was heated from 40–600 °C at a scan rate of 10 °C/min under a constant N_2_ flow of 50 mL/min. The temperature at which 5% weight loss is observed (*T*_id5%_), the onset temperature of main degradation (*T*_d50%_), and residue at 600 °C (%) were recorded from TGA analysis.

### 2.4. Characterization of Film Properties

Fabricated film specimens were thoroughly characterized using DSC for their *T*_g_, *T*_m_, cold crystallization temperature (*T*_cc_), cold crystallization enthalpy (ΔH_c_ in J/g), and the melting enthalpy (ΔH_m_ in J/g). Only, the first heating thermogram (40 to 300 °C) of DSC was recorded to study the effect of stretching on the thermal properties of the films. The degree of crystallinity (*X*_c_) of all the film specimens was also calculated from XRD analysis by the integration of corresponding amorphous and crystalline peaks. For accurate results, fabricated films were analyzed four times. *T*_g_ was estimated as the onset point in heat capacity associated with a transition.

The dimensional stability of the film specimens was determined using a thermo-mechanical analyzer (TMA6100, Seiko Exstar 6000, Seiko Instrument Inc., Chiba, Japan). For this, the values for the coefficient of linear thermal expansion (CTE) of the film specimens (60 × 5 × 0.1 mm^3^) were recorded from 30 to 120 °C with a heating rate of 5 °C/min and analyzed.

The influence of the stretching on the crystalline structure of the films was analyzed by high-power wide-angle X-ray diffraction (XRD, D8 Advance, Bruker Cooperation, Billerica, Massachusetts, United States) spectroscopy. For this, spectrograms of film specimens were obtained using CuKα radiations (λ = 0.154 nm) in the 2θ range of 20 to 50° with a scanning speed of 2θ/min. Tensile testing of the developed samples was carried out using a universal testing machine (UTM, E3000, E3000, Instron, Norwood, Massachusetts, USA), with a crosshead speed of 50 mm/min. Samples were prepared (50 mm × 5 mm) according to the standard test method, ASTM D882 [26], before the testing. For the reliability and accuracy of the mechanical data, the film specimens with different draw ratios (λ) were analyzed four times. The tensile strength was measured as the ultimate tensile strength at the fracture point while Young’s modulus was measured from the initial slope of the S-S curve (strain < 0.1%). The water barrier (%) property was determined by the cup method by following the standard test method ASTM D570-98 [27]. Before the testing, the film samples were conditioned for 1 week at 23 ± 2 °C and 65% RH. The film specimens were placed in the in DI water at 23 ± 2 °C and continued to record the values for the wet weight until they reached a steady-state, usually after 11–12 h.

The water absorption (%) of samples was determined using the wet weight (g) and dry weight (g) data as follows:

Water absorption (%) = [Wet weight (g) − Dry weight (g)] ÷ Dry weight (g) × 100%

Optical properties: transmittance and birefringence (∆n) of fabricated films were determined using a UV–Vis spectrophotometer (S-400, Scinco, Seoul, Korea) and a wide range 2-D birefringence analyzer system (WPA-100-L, Photonic Lattice Inc., Sendai, Japan), respectively. The percentage transmittance (%) for wavelengths from 300–800 nm was recorded and analyzed. For ∆n measurement, retardation of white light in the absence of any sample between the light source and detector was used as a reference. The retardation of the film sample was measured against the reference and analyzed. The ∆n values of film specimens were determined from their retardation (R) and thickness (d) values using the formula as follow:

∆n = R/d

## 3. Results and Discussion

### 3.1. Actual Composition of PCITN Copolyester

**Figure 1:**
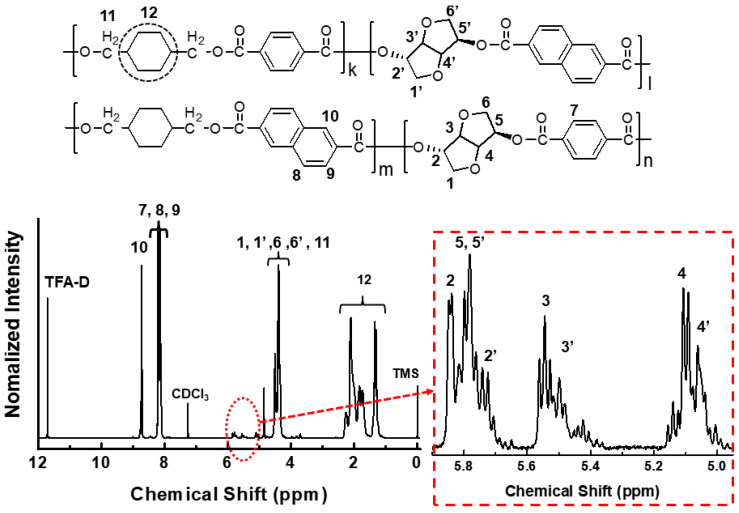
^1^H-NMR spectra of PCITN copolyester.

The composition of monomers: ISB, CHDM, TPA, and NDA was estimated from the integration of peak intensities of each proton of each monomer. The actual chemical composition of synthesized PCITN copolyester was determined by ^1^H-NMR spectroscopy. ^1^H-NMR and assignments of characteristic chemical shifts (δ) (ppm) are shown in Figure 1. Characteristics δ (ppm) peaks at 4.60, 2.12, 1.95, and 1.35 are assigned to trans-CHDM. While δ (ppm) peaks at 4.70, 2.25, 1.83, and 1.70 ppm are assigned to cis-CHDM isomers. δ (ppm) peaks observed at 4.52, 5.84, 5.54, 5.10, 5.80, and 4.36 ppm are assigned to hydrogen atoms 1, 2, 3, 4, 5, and 6 of the ISB linked with terephthalic units. The peaks for 1 and 6 overlap with the hydrogens (11) of the CHDM moiety. δ (ppm) peaks observed at 4.50, 5.73, 5.50, 5.06, 5.78, and 4.34 ppm are assigned to hydrogen atoms 1′, 2′, 3′, 4′, 5′, and 6′ of ISB between the naphthalic unit and the terephthalic unit. The peaks for 1′ and 6′ overlap with the hydrogens (11) of the CHDM moiety.

The δ peaks at 3.40~3.81 ppm are assigned to protons of CH_2_OH at the chain end of the polymer chain. The δ peaks of diacid moieties: TPA (7) and NDA (8 and 9) appear at 8.11~8.21 ppm. However, the δ peak at 8.73 ppm represented by 10 in the figure is assigned to the hydrogen atom of NDA, which enables us to calculate the actual chemical composition of diacid moieties present in PCITN copolyester. The amount of each monomer in PCITN copolyester was determined by the integration of their corresponding δ peaks. Analyzed results (Table 1) indicate synthesized PCITN has a higher amount of CHDM and TPA than feeding monomers, which indicates that CHDM and TPA have higher reactivity than isosorbide and NDA, respectively. Our findings are also supported by the previous research works [28,29,30].

### 3.2. Mechanical Behavior of PCITN Films

**Figure 2:**
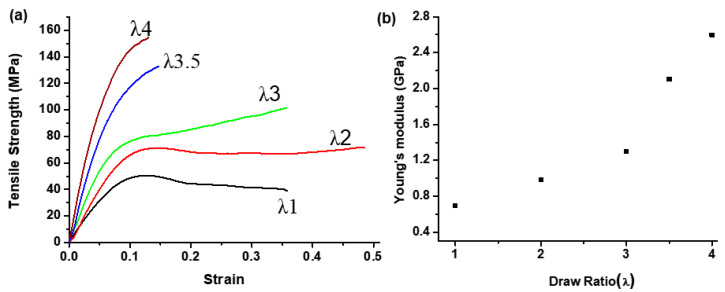
Mechanical properties of cold-drawn PCITN films: (**a**) Tensile strength and (**b**) Young’s modulus as a function of draw ratio (λ).

Tensile testing of the film specimens with different draw ratios (λ) was conducted and the representative stress-strain (S-S) curves are shown in Figure 2. PCITN films showed high tensile strength, as can be seen in Figure 2a. The mechanical properties of as-synthesized PCITN film (λ1) show the ultimate tensile strength as 50.5 MPa and strain as 35.8%. In the case of films having different stretching (λ) such as λ2, λ3, λ3.5, and λ4, the values for the tensile strength are 71.2, 101.5, 132.8, and 154.5 MPa, respectively. It is obvious from the results that as the λ is increased, both tensile strength (MPa) and Young’s modulus (GPa) are also increased (Figure 2b). Initially, tensile strength increased linearly with increasing stretching (λ1–λ3) due to an increment in the molecular orientation of the polymeric chain along the stretching direction. Then, a rapid increment in tensile was observed at higher stretching (λ3–λ4) due to the stress-induced crystallization (SIC). Additionally, the film with the highest stretching (λ4) exhibited the lowest strain at break. It can also be seen from the S-S curve that a decrement in the strain at break is observed with increasing λ. These findings can be attributed to the higher degree of molecular orientation during the stretching of the films. Lee et al. and Hoik et al. also found that mechanical properties of the polymeric materials are directly influenced by the molecular orientation [31,32]. Moreover, the unique V-shaped structure of ISB with a 120° angle between the rings causes the hindrance of polymerization of polymer chains [33]. So, mechanical performance can be affected by the orientation of polymeric chains.

### 3.3. Direct Evidence of SIC by High Power X-ray Diffraction (XRD) Analysis

**Figure 3:**
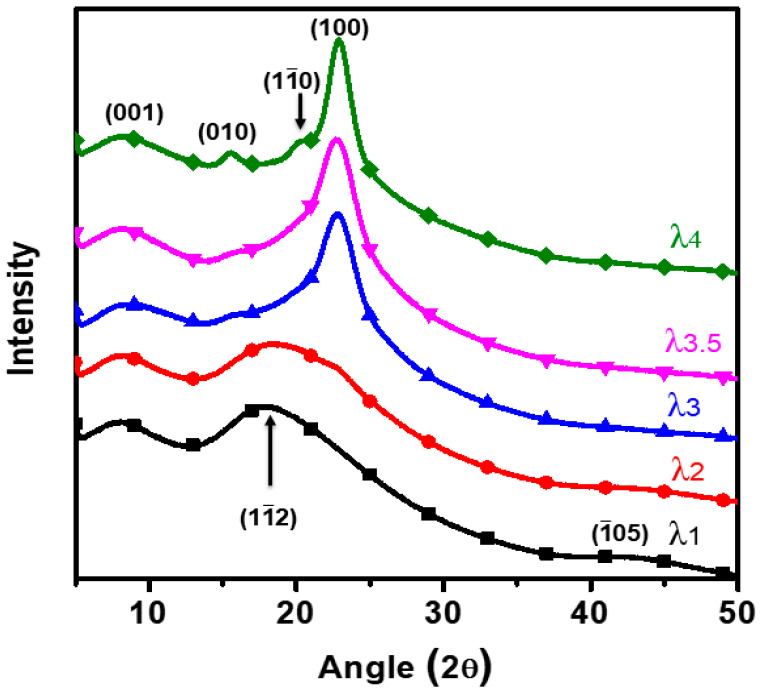
X-ray diffraction pattern of PCITN films stretched at different draw ratios (λ).

The XRD spectrum of the developed PCITN film specimens at the ambient room temperature from 5 to 50 is shown in Figure 3. Three diffraction peaks for the randomly oriented PCTIN film with λ1 that appeared at 7.84°, 18.72° and 42.90° are corresponding to the Miller indices of (001), (1$\bar{1}$2) and ($\bar{1}$05), respectively. The peaks observed at 18.70° and 42.92° are the characteristic peaks of amorphous regions, while the peaks observed at 7.84° are corresponding to the crystalline regions of undrawn (DR1) film. This indicates that as-synthesized PCITN film is semi-crystalline, and it has relatively low crystalline regions compared to stretched films. However, based on the XRD analysis, the reduction in amorphousness of PCITN film can be seen as the intensity of the amorphous peaks decreases with increasing λ. The new peaks are observed at 15.76°, 17.68°, 20.12°, and 23.08° at higher stretching (λ > 3), indicating that at the microscopic level the regular arrangement of molecular chains of the films increases with increasing stretching. These peaks are attributed to the new crystallites that appeared due to stretching and they are attributed to the Miller indices of (010), (1$\bar{1}$1), (1$\bar{1}$0), and (100), respectively [34,35]. It indicates the appearance of the new crystalline regions at the expense of amorphous regions due to the SIC at higher stretching (λ > 3).

### 3.4. Thermal Properties and SIC Analysis of PCITN Film

**Figure 4:**
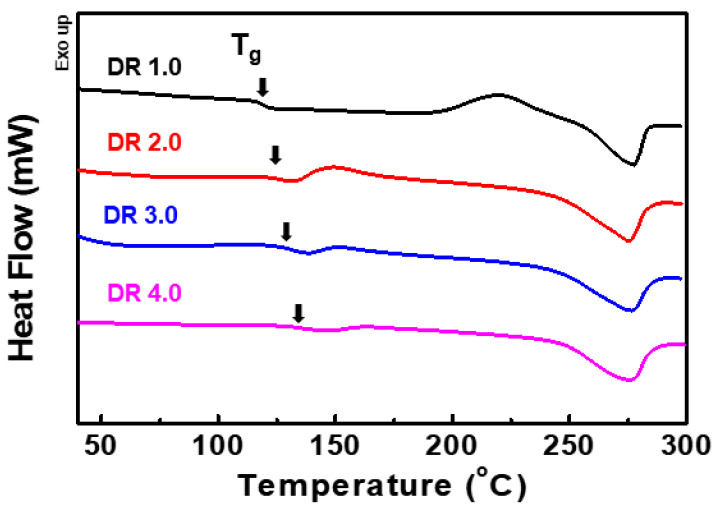
DSC thermograms of the fabricated PCITN films.

The unique chemical structure of ISB with high thermal stability and low segmental mobility makes it an interesting building block for a polymer having high thermal properties, especially the *T*_g_. Thermal properties of the developed uniaxially stretched PCITN films with different λ were analyzed (Figure 4), and the results are summarized in Table 2. It is obvious from DSC analysis that the thermal properties and *X*_c_ are directly influenced by the λ of the film. *T*_g_ was improved linearly while both *T*_cc_ and ΔH_cc_ were reduced with increasing λ. The gradual reduction in ΔH_cc_ indicates that thermally induced crystallites are reduced, and stress-induced crystallites are increased with increasing λ. ΔH_m_ (J/g) and *X*_c_ (%) also confirm the appearance of SIC at higher stretching (λ ˃ 3). Initially, the thermal properties and crystallinity of the stretched films increase linearly with λ due to the gradual growth of molecular chains along the stretching direction. Then, a rapid increment is observed at higher stretching, which can be attributed to new crystallites due to SIC. The effect of the stretching (λ) on thermal and *X*_c_ (%) behavior observed in this study can be due to increased symmetry of polymeric chains, which was improved significantly by stretching. The beauty of this analysis is that its findings are compatible with the previously reported research [36,37,38].

### 3.5. Thermal Stability of the Fabricated PCITN Films

**Figure 5:**
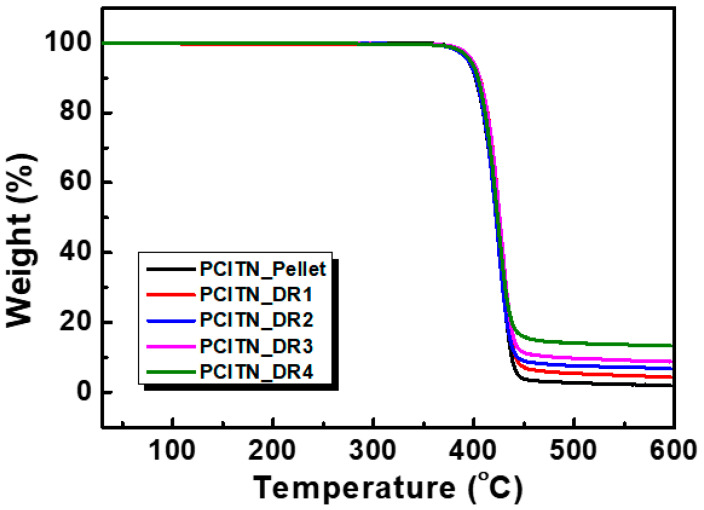
TGA thermograms of the fabricated PCITN films.

Thermal degradation behavior of the fabricated PCITN films stretched at different λ were determined using TGA and the corresponding thermograms, indicating the high thermal stability, are shown in Figure 5. The temperature corresponding to 5% wt loss of the initial weight and residue (%) at 600 °C were recorded from TGA thermograms, and the results are summarized in Table 2. It is evident from the TGA thermograms that all the analyzed samples have a one-stage decomposition, indicating that they have a random copolymer structure. The residue (%) at 600 °C is increased with increasing λ due to increased crystallinity (%) of the resultant stretched polymeric films. The same finding was reported by Um et al. [39]. The high thermal stability of the fabricated film makes it suitable for applications where high thermal stability is required.

### 3.6. Improving the Dimensional Stability of Uniaxially Oriented Films

**Figure 6:**
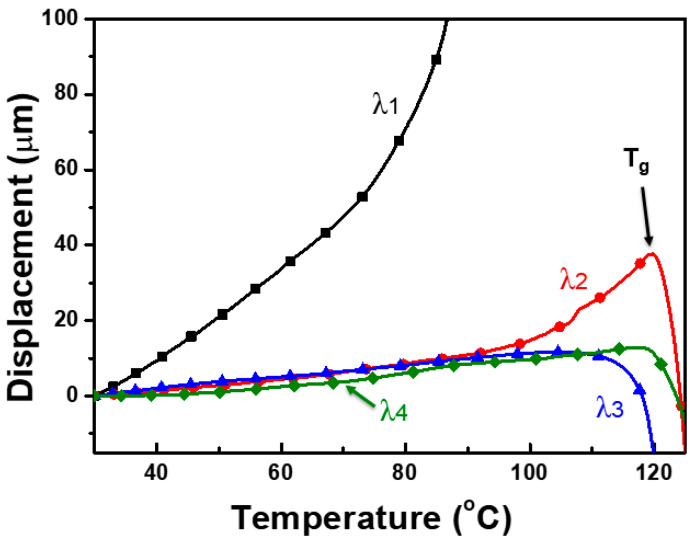
The coefficient of linear thermal expansion (CLTE) plots between 30–120 °C for the uniaxially stretched PCITN films.

Dimensional stability is one of the most important parameters of polymer films for their various industrial applications, especially in the field of flexible electronic devices. The dimensional stability of the fabricated films was determined as their CTE behavior, and the results are shown in Figure 6. It is found that the thermal stability of the PCITN film improves with increasing λ. The least thermal expansion of PCITN film with λ4 indicates that it has the highest dimensional stability, which is due to the higher degree of molecular orientation in this film than other films. Just like conventional plastic films, novel PCITN films also show an undesirable dimensional change around *T*_g_. This phenomenon can be attributed to the facts: (a) the molecular relaxation due to the increased mobility of polymer chains and (b) a rapid shrinkage or expansion due to the residual frozen stress within the stretched film structure. Compared to stretched films, the unstretched film expands abruptly around its *T*_g_ due to increased mobility of molecular chains. CTE behavior of the PCITN films was improved after uniaxial stretching. It can be attributed to the fact that the orientation of molecular chains is significantly improved with increasing λ. It is also reported by the previous researches that that increased molecular orientation improves the dimensional stability of the resultant films [40,41].

### 3.7. Water Barrier Property

**Figure 7:**
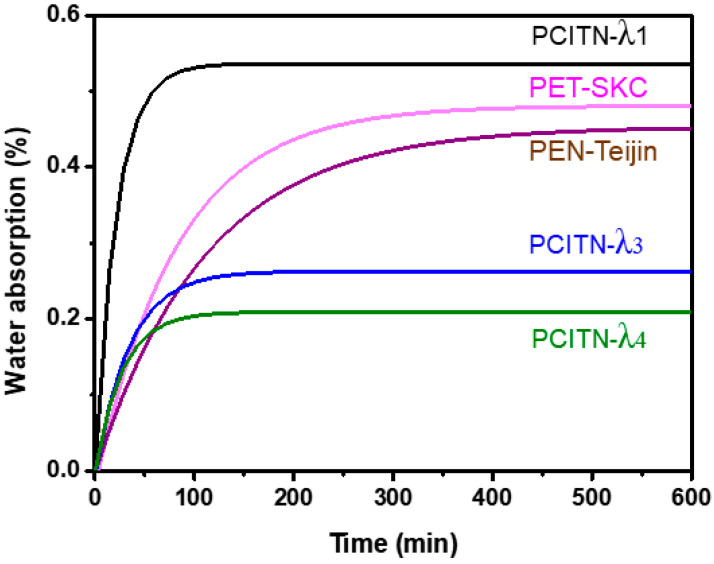
Comparison of the water absorption (%) of developed PCITN films and commercial PET and PEN homopolyester films.

It is well known that good barrier properties of smart polymeric films are critical for their industrial applications especially for next-generation flexible electronic applications. The results for the water barrier of fabricated PCITN in comparison with commercial PET and PEN films are shown in Figure 7. It shows that PCITN film has a better water barrier compared to conventional homopolyester films. This superior water barrier is due to the presence of rigid ISB and cycloaliphatic CHDM units present in the synthesized PCITN films compared to PET and PEN films. This superior water barrier is due to the presence of rigid ISB and cycloaliphatic CHDM units present in the synthesized PCITN films compared to PET and PEN films. It is also obvious from Figure 7 that water barrier property is also improved with increasing λ. The increased water barrier of PCITN film with increasing λ is due to the well-known facts of the improved orientation of polymeric chains and the appearance of new crystallites due to SIC at higher stretching. It is important to note that our findings of water barrier properties are also supported by XRD and DSC results (Section 3.3 and Section 3.4). It is important to note that our findings of water barrier properties are also supported by XRD and DSC results (Section 3.3 and Section 3.4).

### 3.8. Birefringence of As-Synthesized and Uniaxially Stretched PCITN Films

**Figure 8:**
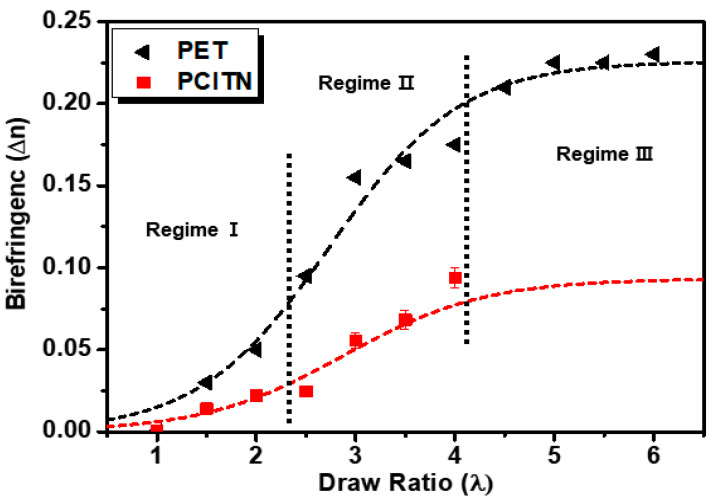
Influence of uniaxial stretching on the birefringence of PCITN films in comparison with uniaxially stretched PET film.

The birefringence can be used to evaluate the molecular orientation and relaxation phenomena of the polymer films. To analyze the orientation development during uniaxial cold-drawing of PCITN films, birefringence has been measured for drawn PCITN films, and the results in comparison with uniaxially stretched PET film [37] are shown in Figure 8. It can be seen from the results (Figure 8) that like PET film, PCITN film also exhibits three regimes during stretching. In the first regime, for λ values up to 1.0~2.5, birefringence increases linearly with increasing λ. In regime II, for λ values up to 3.0~4.0, a rapid increment in the birefringence is observed. In regime III (λ > 4), birefringence reaches its saturation where further stretching cannot cause a significant variation in birefringence. The birefringence results indicate how the molecular orientations are developed during the uniaxial stretching of PCITN. In regime I, the birefringence of isotropic and randomly-oriented films increases linearly with increasing λ due to the steady growth of molecular chains along the stretching direction. An abrupt increment of birefringence in regime II is attributed to the development of SIC phases and the reduction of amorphousness of the stretched films. In regime III, birefringence reaches its saturation due to the achievement of maximum alignment polymer chains. Our results for the birefringence are also supported by the previous research works [37,42]. Our research indicates that novel PCITN film with lower birefringence can be used as an alternative performance material to conventional PET and PEN films for optical applications (PEN > PET > PCITN) [43].

It is worthy to note that the analyzed properties of the developed PCITN film are superior or comparable to the conventional polymeric substrates for flexible electronic devices (Table 3) [20,37,44,45,46]. Uniaxially stretched PCTIN has better optical (Yellow color vs transparent) and water barrier properties (water absorption; 1.8% vs 0.20%) than PI film [47]. Compared to conventional polymer (PET, PEN, and PI), fabricated semi-crystalline PCITN film has unique performance characteristics such as high *T*_g_, wide processing window, good thermal degradation behavior, low CTE, good water barrier, and low birefringence, which make this novel smart film an ideal substrate for next-generation flexible electronics.

The analyzed performance properties of PCITN can be further improved by heat setting and biaxial stretching. The availability of raw materials, ease of synthesis, thermal stability, and wide processing window of PCITN make it suitable for scale-up at the industrial scale. So, it is very easy to develop PCITN film on a commercial scale by adopting the reported methods.

## 4. Conclusions

A novel PCITN copolyester containing a biobased monomer, isosorbide, with a high *T*_g_ and wide processing window was synthesized by melt polymerization. The unique structure of ISB increased the rigidity and *T*_g_ of synthesized copolyester. PCTIN films were successfully fabricated and thoroughly characterized for their thermal, mechanical, optical, and barrier properties after the uniaxial cold drawing (λ = 1~4) at optimized conditions. The performance properties of PCITN were significantly improved due to the increase in molecular chain orientation with increasing λ. The good thermal behavior, high strength, good dimensional stability, good barrier, and optical properties are very useful for polymers as a smart film for industrial applications. The unique characteristics of PCITN compared to conventional polymers make it at futuristic performance material for flexible devices.

## Figures and Tables

**Table 1:** Characteristics of synthesized PCITN copolyester pallets

*T*_g_ (°C) ^a^	123.3	*M*_n_^b^	25,400
*T*_m_ (°C) ^a^	287.4	*M*_w_^b^	68,900
T_cc_ (°C) ^a^	222.9	PDI (*M*_n_/*M*_w_) ^b^	2.70
ΔH_m_ (J/g) ^a^	46.99	IV ^c^ (dL/g)	0.77
CHDM/ISB Feed Ratio	75/25	CHDM/ISB Composition Ratio ^d^	93.92/6.08
TPA/NDA Feed Ratio	25/75	TPA/NDA Composition Ratio ^d^	30.95/69.05

^a^ Glass transition temperature (*T*_g_), cold crystallization temperature (*T*_cc_), melting temperature (*T*_m_), and melting enthalpy ΔH_m_ (J/g) were determined by differential scanning calorimeter (DSC). ^b^ Number average molecular weight (*M*_n_, g/mol), weight average molecular weight (*M*_w_, g/mol), and polymer dispersity index (PDI) were determined by gel permeation chromatography (GPC). ^c^ The intrinsic viscosity (IV) was determined in o-chloroform (OCP) solvent by using automated Ubbelohde viscometer (no. 1C) at 30 °C. ^d^ Actual chemical composition of each monomer in PCITN was determined by proton nuclear magnetic resonance (^1^H-NMR) spectroscopy.

**Table 2:** Influence of stretching on the thermal properties and *X*_c_ of PCITN film.

Draw Ratio	*T*_g_ (°C)	*T*_cc_ (°C)	ΔH_cc_ (J/g)	*T*_m_ (°C)	ΔH_m_ (J/g)	*X*_c_ (%)	*T*_id5%_ (°C)	Residue at 600 °C (%)
1	123.9	220.5	18.20	277.5	20.49	8.65	394.7	1.9
2	125.1	149.2	10.61	275.1	21.86	43.43	398.3	4.3
3	131.9	-	-	276.1	25.32	75.92	398.4	8.7
4	134.9	-	-	275.4	26.66	97.65	396.4	13.4

**Table 3:** Characteristics of uniaxially stretched PCITN film in comparison with PET, PEN, and PI films; conventional polymeric substrate for flexible electronics

Property	PET (Melinex)	PEN (Teonex)	PI (Kapton)	PCITN (Uniaxially Stretched)
*T*_g_ (°C)	78	120	360	140
*T*_m_ (°C)	260	268	-	275
Commercial Availability	Yes	Yes	Yes	No
Transmission (400–700 nm), %	89	87	Yellow	86
CTE (−55 to 85 °C) (ppm °C^−1^)	15	13	30–60	5.8
Young’s modulus (GPa)	5.3	6.1	2.5	2.6
Birefringence (△n) *(Uniaxially Oriented)*	0.24	0.48	-	0.09
Water absorption (%) *(Randomly Oriented)*	1.08	0.98	-	0.54
